# Quality Improvement in Canadian Nephrology: Key Considerations in Ensuring Thoughtful Ethical Oversight

**DOI:** 10.1177/20543581221077504

**Published:** 2022-02-27

**Authors:** Tamara Glavinovic, Jay Hingwala, Claire Harris

**Affiliations:** 1 Division of Nephrology, Department of Medicine, Sunnybrook Health Sciences Centre, University of Toronto, ON, Canada; 2 Division of Nephrology, Department of Medicine, Health Sciences Centre, University of Manitoba, Winnipeg, Canada; 3 Division of Nephrology, Department of Medicine, Vancouver General Hospital, University of British Columbia, Canada

**Keywords:** ethics, quality improvement, quality assurance, ethics approval

## Abstract

**Purpose of review:**

Quality improvement (QI) work is a cornerstone of health care, and a growing area within nephrology. With such growth comes the need to ensure that QI activities are implemented in an ethically responsible manner. The existing institutional research board (IRB) framework has largely focused on reviewing the ethical suitability of traditional research projects, and it can be challenging to know if QI initiatives require formal ethics oversight. Several tools have been developed to assist in distinguishing between the two, such as the “A pRoject Ethics Community Consensus Initiative” tool. Our objective was to demonstrate how QI is distinct from research, to outline how QI-focused IRB process is used across Canada, and to develop a practical aid for clinicians embarking on QI-related projects.

**Sources of information:**

Publicly available institutional Web sites from academic and select nonacademic sites across Canada.

**Methods:**

Institutional Web sites across all academic centers within Canada were examined to determine local QI-specific ethics review processes. We have provided examples of QI processes from select community sites. We have developed a tool to assist clinicians navigate the ethical challenges of QI projects and to determine whether their project may require ethics approval.

**Key findings:**

This overview of the considerations of the research ethics approval process helps clinicians to determine whether IRB approval is required for QI studies. Examples of the current ethical processes employed in both academic and community institutions across Canada demonstrate the variability between centers. We have included examples of fictional nephrology-oriented QI initiatives to illustrate when ethics approval may be considered, along with a flowchart. This summary highlights the opportunity for QI-specific IRB review processes to be standardized across Canada, along with the need for creation of a separate stream with dedicated expertise for QI project review.

**Limitations:**

We did not do a formal environmental scan of the QI IRB review process in all hospital institutions across Canada.

## Abrégé

**Justification:**

Les travaux visant l’amélioration de la qualité (AQ) sont une des pierres angulaires des soins de santé. L’AQ est un secteur en croissance en néphrologie et avec une telle croissance vient la nécessité de s’assurer que les activités d’AQ sont mises en œuvre de manière éthique et responsable. Le cadre actuel des comités d’éthique de la recherche (CER) s’est essentiellement concentré sur l’examen de la pertinence éthique des projets de recherche traditionnels, et il peut être difficile de savoir si les initiatives d’AQ nécessitent une surveillance formelle de l’éthique. Plusieurs outils ont été mis au point pour faciliter la distinction entre les deux, notamment l’ARECCI. Notre objectif était de démontrer en quoi l’AQ se distingue de la recherche, d’indiquer dans quelle mesure les processus des CER sont axés sur l’AQ à travers le Canada et de développer une aide pratique pour les cliniciens qui se lancent dans des projets relatifs à l’AQ.

**Sources:**

Des sites institutionnels accessibles au public provenant de sites universitaires et de certains sites non universitaires à travers le Canada.

**Méthodologie:**

Les sites Web institutionnels de tous les centers universitaires du Canada ont été examinés afin de déterminer les processus locaux d’examen de l’éthique propres à l’AQ. Nous avons fourni des exemples de processus d’AQ provenant de sites communautaires sélectionnés. Nous avons mis au point un outil pour aider les cliniciens à relever les défis éthiques des projets d’AQ et à déterminer si leur projet pourrait nécessiter une approbation éthique.

**Principaux résultats:**

Cet aperçu des éléments à considérer dans le processus d’approbation de l’éthique de la recherche aide les cliniciens à déterminer si l’approbation du CER est requise pour les études d’AQ. Les exemples des processus d’examen de l’éthique qui sont actuellement employés dans les établissements universitaires et communautaires du Canada démontrent la variabilité entre les centers. Nous avons inclus un diagramme de flux et des exemples d’initiatives fictives d’AQ axées sur la néphrologie pour illustrer les cas où l’approbation de l’éthique peut être envisagée. Ce résumé met en évidence la possibilité d’uniformiser les processus d’examen des CER propres à l’AQ dans l’ensemble du Canada, ainsi que la nécessité de créer un volet distinct doté d’une expertise dédiée à l’examen des projets d’AQ.

**Limites:**

Nous n’avons pas procédé à une analyze environnementale officielle du processus d’examen de l’AQ par les CER de tous les établissements hospitaliers du Canada.

## What was known before

Quality improvement (QI) is a developing field within nephrology and is integral to improving and providing excellent care. Determining whether QI initiatives constitute research necessitating institutional research board (IRB) approval is not always simple, with multiple factors to consider.

## What this adds

This overview of the considerations of the research ethics approval process helps clinicians to determine whether IRB approval is required for QI studies. This work highlights how IRB practices differ across Canada. We have also created a series of fictional nephrology-oriented QI initiatives to illustrate when ethics approval may be considered, and we provide a practical tool to help guide project leaders.

## Introduction

Quality improvement (QI) and quality assurance (QA) work are the cornerstones of ongoing improvement within health care.^1-3^

Nephrology provides fertile ground for QI initiatives as much of the care provided by nephrology teams is multidisciplinary and highly systems-based. In recent years, there has been a growing focus on developing the scope of QI work within nephrology. This has ranged from a national initiative to categorize and prioritize quality indicators currently in use,^4-7^ to a growing body of literature providing guidance on the development of QI initiatives in improving aspects of kidney care,^8-12^ to various local QI initiatives.^13-15^

With this growth comes the need to ensure that QI activities are implemented in an ethically responsible manner, and that we have systems of oversight in place that can thoughtfully and efficiently provide direction on important ethical considerations.

It can be a challenge to determine whether QI initiatives require ethical oversight by a formal institutional research board (IRB). Ethics approval within QI work is a developing field and there are key differences in scope and methodology between QI and traditional research,^16-18^ and these differences have created ambiguity regarding when IRB approval should be sought and when it may not be necessary.^19,20^ Our objective was to demonstrate how QI is distinct from research, to outline how QI-focused IRB is used across Canada, and to develop a practical aid for clinicians embarking on QI-related projects.

## Methods

We reviewed publicly available institutional Web sites from all academic centers across Canada to determine their local IRB processes. Academic institutions where no information was publicly available were also identified. We included examples of the ethics review process from select community sites across Canada. We have created a series of fictional clinical cases to illustrate the ethical considerations specific to each proposed quality initiative and we have created a clinical aid for clinicians to use when starting a QI initiative to help them determine whether ethics review is required.

## Review

### How Is QI Different From Research?

Quality improvement work and research are not always easily distinguishable and may share similar attributes. Quality improvement as a matter of routine practice to improve local health care delivery can sometimes be considered distinct from research; however, there may be significant overlap. Quality improvement refers to activities that are designed to improve health care in a particular setting, such as the processes of providing care, or reviewing patient outcomes. This encompasses QA, which aims to assess if existing care is adequate.^21-23^ Quality improvement may create new information that could be used by others outside of the local institution, though by nature of its focus on local practice, it is less likely to be considered widely generalizable.^18^ An assumption of QI-related work is that all who receive care as a part of these QI projects or initiatives will benefit^24^ and QI work focuses on the implementation of these initiatives. As well, QI initiatives are assumed to create no more than minimal risk to participants (health care provider or patient),^25^ as they generally do not involve experimental therapies but rather standard of care.^25^ Additionally, QI-related work often collects aggregate data that does not require collecting patient identifiers.

Pragmatic research, on the other hand, allows us to develop and identify which of these interventions will be most effective in a clinical setting and which would be best for implementation of a process of care.^18^ As a result, findings from pragmatic research studies may be generalizable outside of a given setting and of great interest to other centers in contrast to QI studies. Casarett et al. proposed 2 criteria to help identify whether a project should be considered research. The first suggested that if the majority of patients were not “expected to benefit directly from the knowledge to be gained,” this should be considered research. The second criteria focused on there being additional risks or burden to participants that may be imposed in order to create generalizable results^16^ in traditional research. However, additional characteristics of a study need careful consideration for ethical oversight, such as its funding source,^26,27^ explicit elements of research such as human participants or blinding aspects of care, differing treatment groups or changes from standard of care, collection of personal information, use of databases to extract information, or if there is a predetermined plan to publish and disseminate the work. Notably, none of these criteria negate the need for health care providers conducting QI projects to follow ethical principles in the conduct of QI work regardless of a formal requirement for approval.^28^

### Why Should Ethics Approval Be Considered? Are There Barriers to Seeking IRB Approval?

There are both advantages and challenges associated with seeking ethics oversight. While it is prudent for institutional ethics boards to ensure that those with QI expertise and familiarity with the methodologies reviewing QI projects, this may not be available within existing IRB infrastructures. There are instances when QI initiatives may have features of both QI and research, and it may be unclear whether IRB approval should be sought. Ethical oversight may serve as a helpful resource to ensure that the methodology of a project is sound. Ethical oversight, whether via a formal ethics review process or that which is supervised by local QI officers, is also important to ensure that the interests of providers and patients are protected, that there is minimization of health care waste, and that confidentiality is respected. Data confidentiality must be ensured, particularly if there is multicenter or multisite collaboration on a particular project, and IRB review serves as a check stop in this regard.

Formal IRB review can pose some challenges as traditional research follows a strictly adhered-to finalized design and process. If changes are required after IRB approval, there must be resubmission for additional review. Quality improvement initiatives rely on an iterative design known as the “Plan-Do-Study-Act” model which must be nimble and flexible to adapt to changes and to react to findings noted during earlier stages of implementation.^29^ Research ethics submission and approval can be a lengthy process and having to resubmit a proposal to the ethics board at each stage of iterative change would be unduly burdensome both for those involved in the QI initiatives and for the committees responsible for ethical oversight. This requirement would potentially dissuade individuals from embarking on local QI initiatives. As a result, several tools have been developed to provide guidance on how to approach and determine the need for IRB review.

### What Is the Tri-Council Policy? Who Can Provide Ethical Oversight for QI Projects?

The tri-council policy was developed by the government of Canada to establish principles that would serve to guide ethical conduct for research involving humans. Specific to QI, the tri-council policy serves as an aid to determine when ethics approval is required and/or recommended for QI or QA studies. The policy states that:

Quality assurance and quality improvement studies, program evaluation activities, and performance reviews, or testing within normal educational requirements when used exclusively for assessment, management, or improvement purposes, do not constitute research for the purposes of this policy, and do not fall within the scope of research ethics board review.^17^

However, if that same data which was collected for the above purposes is later used for research (for which it was not originally explicitly intended), it may then require IRB approval.

Ethical oversight can be provided by an already existing research ethics board at certain institutions, by local QI officers, or by a dedicated IRB for QI-related initiatives. There is considerable variation across institutions. Existing research ethics boards may be best suited for oversight for all QI-related projects, as they are already established and have processes in place that are generally understood and accepted. However, a traditional IRB process may not have the necessary specific requisite knowledge to assess projects in a QI lens, to allow for the rapid cycle changes necessary for QI initiatives. Additionally, with the growth of QI work being done within medicine, the requests for review of many new QI-specific proposals may be untenable for these oversight bodies. As such, some solutions include QI-specific review committees that operate on either an institutional or regional level that would work similarly to an IRB, but with the specific focus of QI/QA activities.

### What Policies or Tools Have Been Developed to Help Guide Clinicians?

A number of individual institutions across Canada have made available their local guidelines and screening processes to help local researchers and clinicians decide when ethics approval is required for QI initiatives, and have provided information on how to differentiate these projects from traditional research.^30,31^ These principles of these tools align with the principles set out by the tri-council policy.

The “A pRoject Ethics Community Consensus Initiative” (ARECCI) tool is one such tool that was developed by Alberta Innovates—Health Solutions to provide decision-making support to clinicians embarking on QI-related projects to help navigate the ethical considerations and the necessary ethics oversight.^32^ This tool consists of 6 key ethical considerations summarized in Table 1.

**Table 1.** The “ARECCI” Guide for Ethical Consideration in Quality Improvement.^32,a^

1. How will the knowledge gained from this project be useful?
2. How will the described method or approach generate the desired knowledge?
3. How will you ensure that the participant (or data) selection process is fair and appropriate?
4. What have you done to identify and minimize risks? Are the remaining risks justified?
5. How are the rights of individuals, communities, and populations respected in this project?
6. Is informed consent needed in this project?

*Note.* ARECCI = A pRoject Ethics Community Consensus Initiative.

a The ARECCI tool was developed to help investigators determine the level of risk of a project, the type of risks involved, and the type of ethical review that might be required.

This tool guides clinicians through a series of questions to help determine whether the project should be considered research or QI. If the proposed project is screened as likely to be considered research, the tool then recommends that the project be sent to an ethics board for review. While many institutions may not have a separate review process for QI projects, this stepwise approach can provide access to a streamlined process for abbreviated review and provide a dedicated contact person for questions about whether to pursue a formal IRB review before embarking on any given project. This can help expedite valuable QI projects that, for example, would not be subject to full IRB review.

### Clinical Cases

**Figure 1.**
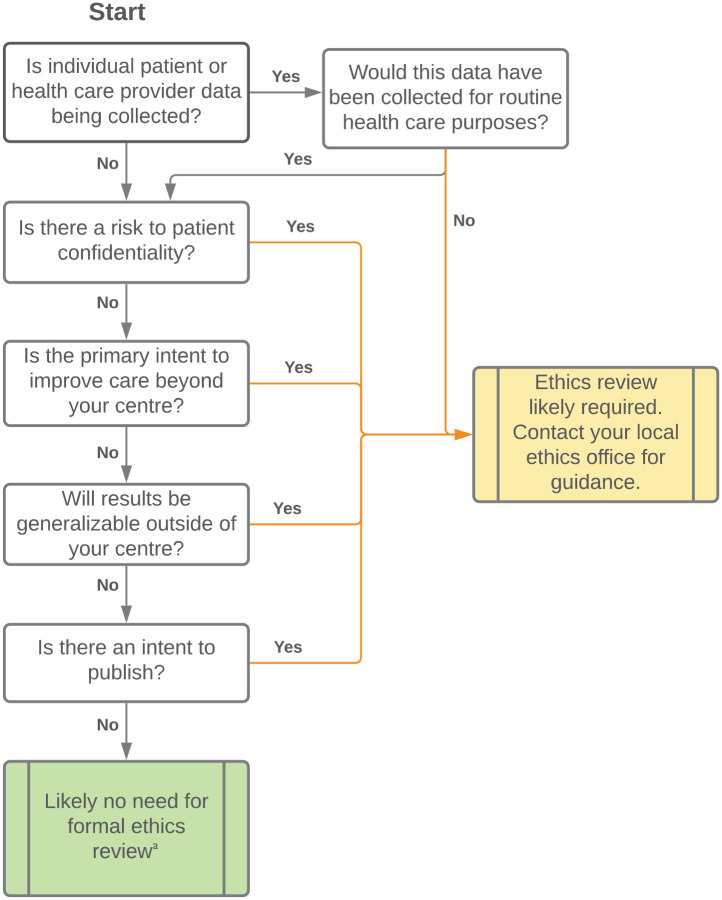
Flowchart to aid clinicians navigate the ethical challenges of QI projects. *Note.* QI = quality improvement. ^a^We encourage project leaders to familiarize themselves with their local policy and to contact their ethics board prior to starting any project. Certain programs may require registration of all ongoing QI projects even if no formal institutional research board is required.

We have created a series of clinical cases relevant to nephrology to illustrate key ethical considerations and the challenge of differentiating whether a project may or may not benefit from ethics oversight. These clinical cases are fictional, with some modified based on the authors’ experiences. These cases illustrate the complexity that exists, and we encourage clinicians to “check-in” with the local IRB team to determine if a waiver or formal review is needed for their individual projects (Table 2). Many of the below-mentioned cases have ethical considerations that require IRB review (either formal or informal). We have also created a flowchart to aid clinicians in navigating the ethical challenges that may arise when embarking on QI projects (please see Figure 1).

**Table 2.** Clinical Cases and Ethical Considerations.

Clinical cases	Ethical considerations	Ethics approval next steps
Your program is hoping to establish a protocol to standardize IV iron infusions for eligible maintenance hemodialysis patients and to compare anemia parameters pre-initiation and post-initiation of this initiative. This QI initiative is not externally/internally funded and will not be using an experimental IV iron formulation (ie, adhering to current standard of practice). Data will need to be collected on patient variables and anemia parameters prior to and after implementation of this protocol. The primary goal of this initiative will be to improve care within your organization, and while possibly publishing this in a journal as a secondary goal.	- Data confidentiality and risk to patients - Primary vs secondary goal of publication	If data being collected is already routinely collected, this may not constitute additional risk to patients involved. With the main goals being standardizing care among patients to current standard of care, this is more likely to quality as QI /QA and may not require full ethics approval. However, while the goal of publishing this is secondary, certain institutions across Canada may consider this as a criteria for formal ethics approval. Checking in with your local ethics board is encouraged.
You want to distribute a survey to health care providers about their perceived barriers to delivering dialysis-related care. Data from care providers will be anonymized. You then hope to publish the results of this survey to inform future studies.	- No patient data will be collected, though data from health care providers will be collected that would not be routinely collected - Intent to publish - No external funding	The survey will generate new knowledge and is likely to be considered research. As such, ethics approval will be required for this survey. Depending on local ethics processes, this project may be considered “low risk” and possibly expedited.
You want to survey patients on their perceptions of how care changed with the COVID-19 pandemic. You are unsure if you will publish the results, but you wish to use the data to improve care at your center. Qualitative results obtained will be anonymized and will be collected prospectively.	- Patient data will be used that would not routinely be collected - Consent implied at time of participation - Confidentiality will be maintained via data anonymization - Care will not change directly related to this survey	Since individual patient data will be used that is out of the routine and that this will generate new data which may be generalizable outside of this health care setting, it would be advisable to submit this project proposal for ethics review.
You want to do a randomized trial to change the vascular access lock for dialysis patients. This trial has been co-funded by a grant you have obtained from a national agency. Patients at your hospital-based and satellite units will be randomized to 1 of 2 vascular access locks, both of which are used in practice. You will not be using an experimental technique/drug, but you will be using 2 different medicines and comparing results.	- Risks more than minimal to patients - Is standard of care changing - Patient data will be collected outside of what is considered routine. External funding	We suggest submitting this project for ethics review given the external funding and the generation of new, potentially generalizable knowledge.^26,27^
You want to do an audit to determine the proportion of patients that provide home BP monitoring results during their clinic visits. This information will then be used to develop an educational initiative to encourage the use of home BP results. Results will likely not be generalizable outside of the local setting and unlikely to be published.	- You will be using individual patient data which will not be anonymized. - You will use this data to target certain groups to increase use of home BP monitors.	Since a retrospective chart review will be done utilizing patient data, it would be advisable to submit this proposal to determine if full ethics review is needed. Since the risks are low and the primary intent is to improve care at your center, this project may be classified by your local center as a low-risk project that may only need expedited review or project registration.
You are performing an environmental scan to determine which quality indicators are currently in use across Canada in home dialysis. You will be contacting health care providers across centers and obtaining information from them. You will not be using any patient data, nor will you be affecting day-to-day care. You will then be rating and prioritizing these indicators using an established method to determine which ones are best suited to future QI-related work. Local programs may then choose to use these prioritized indicators to measure quality of care.	- No patient or health care provider data will be collected - Certain information is in the public domain with no expectation of privacy - Intent to publish - No external funding necessitating institutional research board approval	There will be no need for ethics approval given the minimal level of risk that would be experienced by patients and providers as no individual patient or health care provider data is collected and all information is in the public domain. Intent to publish such findings does not necessarily mean that ethics approval is required and discussing with the local ethics board is advisable.

*Note.* QI = quality improvement; BP = blood pressure; IV: intravenous.

## Institutional Review Board Practices Across Canadian Centers

We have provided examples of IRB practices from all academic institutions across Canada in Table 3 where information was publicly available.^31,33-49^ Two academic institutions did not have information available on their Web sites specific to QI (n = 2/18, 11%) (Center hospitalier de l’Universite de Montreal and Universite Laval). Of the remaining 16 academic institutions where information was available, we found that 44% (n = 7/16) institutions employ the ARECCI tool to help clinicians determine whether their project might need IRB review, and 19% (n = 3/16) reference the tri-council policy when outlining which projects require formal review. We found that 38% (n = 6/16) institutions require QI project registration even if no formal ethics review is required, and all institutions recommend contacting the ethics office should there be any uncertainty about whether ethics review is required. None of the ethics institutions specified whether there were members with dedicated QI expertise as part of their formal ethics review process, and none of the programs provided publicly available information regarding how turnaround time is managed for projects which incorporate a “Plan-Do-Study-Act” format that may require rapid alterations to the original proposal.

**Table 3.** Environmental Scan of the Quality Improvement Ethics Review Process Across Academic Centers in Canada.

Institutions	IRB process for QI projects
Memorial University^33^	Questionnaire provided to determine if IRB or separate privacy compliance review required for QI/QA projects
Dalhousie University^34^	• Local guidelines developed based on tri-council policy to help determine if QI projects require IRB review • Intent to publish does not determine if a project is considered research necessitating IRB review If uncertainty exists, investigators are encouraged to contact the ethics office
Center hospitalier de l’Université de Montréal	No information publicly available specific to QI
Université de Sherbrooke	No information publicly available specific to QI
Université Laval^36^	• Projects exempt from IRB approval include those pertaining to QA and program evaluation Must obtain formal exemption from the IRB by submitting full project description
McGill University^35^	• ARECCI questionnaire used to determine if project requires full IRB review • Separate QI project proposal required For use of information within medical records, need authorization from the director of professional services
University of Ottawa^30^	• Guideline document outlining characteristics of QI/QA vs research intended as an aid for clinicians • ARECCI questionnaire used to determine need for IRB review QI/QA projects require registration at the Quality Office
Queen’s University^37^ (includes academic and affiliated community sites)	• ARECCI questionnaire used to determine if project requires full IRB review • Local guidelines developed based on tri-council policy to help determine if QI projects require IRB review If uncertainty exists, investigators are encouraged to contact the ethics office
McMaster University^38^	• ARECCI questionnaire used to determine need for IRB review
Western University^39^	QI project proposals must be filled out and submitted on the local research ethics board Web site to determine if exemption required
University of Toronto^40^	• Hospital-specific IRB review applications ARECCI questionnaire used to determine need for IRB review
Northern Ontario School of Medicine (Laurentian University, Lakehead University, Thunder Bay Regional Health Sciences Center)^41^	• Need to apply for formal waiver if doing a QI project Individuals applying for a waiver must reference the sections of the tri-council policy in their application
University of Manitoba^42^	• Investigators embarking on QI/QA projects must submit a proposal and have written approval of exemption from the local IRB Intent to publish noted as a requirement for IRB approval
University of Saskatchewan^43^	Guideline document outlining differences between QI/QA vs research intended as an aid for clinicians
University of Calgary^44^	• QI/QA project proposals must be formally submitted to obtain an exemption ARECCI questionnaire used to determine need for IRB review
University of Alberta^45^	• Guideline document outlining differences between QI/QA vs research intended as an aid for clinicians IRB review encouraged if uncertain of category
University of British Columbia (Vancouver Coastal Health, Providence Health Care—academic and affiliated community sites)^31^	• Document outlining differences between QI/QA and research with explanation of terms ARECCI questionnaire used to determine need for IRB review
Nunavut Research Institute (includes affiliated community sites)^46^	• Joint consideration of QI/QA projects is needed between the Nunavut Research Institute and affiliated tertiary care centers based in Winnipeg, Edmonton, Ottawa QI/QA projects using Nunavut residents’ health information require confirmation that need for full IRB review has been waived by the affiliated tertiary care center
Select Examples of Community Hospitals^a^	
William Osler Health System^47^	• No information publicly available specific to QI Delegated reviews (vs full review) are conducted for minimal-risk, noninvasive studies (eg, retrospective chart reviews, questionnaires, surveys, etc)
Lakeridge Health	• No information publicly available specific to QI
Humber River Hospital	• No information publicly available specific to QI
Winnipeg Regional Health Authority (includes academic and community sites)^48^	• QI project proposals must be submitted to determine if full IRB review is needed

*Note.* IRB = institutional research board; QI = quality improvement; QA = quality assurance; ARECCI = A pRoject Ethics Community Consensus Initiative.

a Community sites with academic affiliation may be required to undergo IRB review aligning with the policies of the affiliated academic center.

We have also provided select examples of the IRB process at several community sites across the country in Table 3. Certain community sites were found to be under the umbrella of the affiliated academic center (eg, Winnipeg Regional Health Authority, Vancouver Coastal Health/Providence Health, Queen’s University) and follow their respective IRB processes. Opportunity exists for future studies to examine the variability in barriers to implementing innovative QI projects between community and academic sites, as little is currently known in this area.

## Limitations

Limitations deserve mention. First, we did not do a formal environmental scan of all medical institutions across Canada. While we included examples from all academic centers where information was available on their institutional Web site, not all academic centers had such information available to the public. Additionally, we do not have representation from all community sites across Canada. Second, we did not contact individual IRBs directly to obtain information related to their ethics processes, their individual experiences with QI projects. We were also unable to ascertain if QI-related expertise was incorporated into the composition of their ethics boards.

## Conclusions

Quality improvement and research are not always easily distinguishable from each other. They employ differing methodologies and often have different aims in terms of either generalizability or desire for publication. In certain instances, there is disagreement between individuals regarding whether IRB approval is necessary. For example, Lindenauer et al. found that a survey of individuals involved in IRB review, quality officers at a hospital, and journal editors had differing views on which projects required IRB approval.^49^ As ethics boards were not originally designed with QI-related work in mind, we encourage the use of the ARECCI tool, Figure 1, and subsequent inquiry with your local ethics board to determine if formal ethics approval is required for any given project. While a separate oversight body may be helpful, if not possible, it may be reasonable to have QI projects be evaluated via an abbreviated and separate ethics approval stream. Even if formal ethics approval is waived, ethical practices to ensure patient confidentiality and data integrity remain critical for any QI project.

## References

[1] America IoMUCoQoHCi. In: Kohn LT Corrigan JM Donaldson MS , eds. To Err is Human: Building a Safer Health System. Washington (DC): National Academies Press (US);2000:17-25. 25077248

[2] *CAmerica IoMUCoQoHCi* . Crossing the Quality Chasm: A New Health System for the 21st Century. Washington (DC): National Academies Press (US); 2001. 25057539

[3] Adams K Corrigan JM . In: Adams K Corrigan JM , eds. Priority Areas for National Action: Transforming Health Care Quality. Washington (DC): National Academies Press; 2003:15-28. 25057643

[4] Dubrofsky L Ibrahim A Tennankore K Poinen K Shah S Silver SA . An environmental scan and evaluation of home dialysis quality indicators currently used in Canada. Can J Kidney Health Dis. 2020;7:2054358120977391. doi:10.1177/2054358120977391. PMC7734484 33354332

[5] Blum D Thomas A Harris C Hingwala J Beaubien-Souligny W Silver SA . An environmental scan of Canadian quality metrics for patients on in-center hemodialysis. Can J Kidney Health Dis. 2020;7:2054358120975314. doi:10.1177/2054358120975314. PMC7727051 33343910

[6] Hingwala J Molnar AO Mysore P Silver SA . An environmental scan of ambulatory care quality indicators for patients with advanced kidney disease currently used in Canada. Can J Kidney Health Dis. 2021;8:2054358121991096. doi:10.1177/2054358121991096. PMC7868503 33614057

[7] Glavinovic T Vinson AJ Silver SA Yohanna S . An environmental scan and evaluation of quality indicators across Canadian kidney transplant centers. Can J Kidney Health Dis. 2021;8:20543581211027969. doi:10.1177/20543581211027969. PMC8243101 34262781

[8] Silver SA Bell CM Chertow GM , et al. Effectiveness of quality improvement strategies for the management of CKD: a meta-analysis. Clin J Am Soc Nephrol. 2017;12:1601-1614. doi:10.2215/CJN.02490317 28877926 PMC5628709

[9] Harel Z Silver SA McQuillan RF , et al. How to diagnose solutions to a quality of care problem. Clin J Am Soc Nephrol. 2016;11:901-907. doi:10.2215/CJN.11481015. 27016495 PMC4858489

[10] Silver SA McQuillan R Harel Z , et al. How to sustain change and support continuous quality improvement. Clin J Am Soc Nephrol. 2016;11:916-924. doi:10.2215/CJN.11501015 27016498 PMC4858491

[11] McQuillan RF Silver SA Harel Z , et al. How to measure and interpret quality improvement data. Clin J Am Soc Nephrol. 2016;11:908-914. doi:10.2215/CJN.11511015. 27016496 PMC4858492

[12] Silver SA Harel Z McQuillan R , et al. How to begin a quality improvement project. Clin J Am Soc Nephrol. 2016;11:893-900. doi:10.2215/CJN.11491015 27016497 PMC4858490

[13] Glavinovic T Kashani M Al-Sahlawi M , et al. A Peritoneal dialysis access quality improvement initiative: a single-center experience. Perit Dial Int. 2019;39(5):437-446. doi:10.3747/pdi.2018.00233. 31123070

[14] Blum D Beaubien-Souligny W Battistella M , et al. Quality improvement program improves time in therapeutic range for hemodialysis recipients taking warfarin. Kidney Int Rep. 2020;5(2):159-164. doi:10.1016/j.ekir.2019.10.011. 32043029 PMC7000800

[15] McIntyre C McQuillan R Bell C Battistella M . Targeted deprescribing in an outpatient hemodialysis unit: a quality improvement study to decrease polypharmacy. Am J Kidney Dis. 2017;70(5):611-618. doi:10.1053/j.ajkd.2017.02.374. 28416321

[16] Casarett D Karlawish JH Sugarman J . Determining when quality improvement initiatives should be considered research: proposed criteria and potential implications. JAMA. 2000;283:2275-2280. doi:10.1001/jama.283.17.2275 10807388

[17] Ethics GoC-PoR. Tri Council Policy Statement 2 (2018)–Chapter 2: Scope and Approach. Government of Canada. https://ethics.gc.ca/eng/tcps2-eptc2_2018_chapter2-chapitre2.html

[18] Finkelstein JA Brickman AL Capron A , et al. Oversight on the borderline: quality improvement and pragmatic research. Clin Trials. 2015;12(5):457-466. doi:10.1177/1740774515597682. 26374685 PMC4699562

[19] Taylor HA Pronovost PJ Faden RR Kass NE Sugarman J . The ethical review of health care quality improvement initiatives: findings from the field. Issue Brief (Commonw Fund). 2010;95:1-12. 20726137

[20] Taylor HA Pronovost PJ Sugarman J . Ethics, oversight and quality improvement initiatives. Qual Saf Health Care. 2010;19(4):271-274. doi:10.1136/qshc.2009.038034. 20511239

[21] Nelson EC Splaine ME Batalden PB , et al. Building measurement and data collection into medical practice. Ann Intern Med. 1998;128:460-466. doi:10.7326/0003-4819-128-6-199803150-00007 9499330

[22] Batalden PB Davidoff F . What is “quality improvement” and how can it transform healthcare. Qual Saf Health Care. 2007;16(1):2-3. doi:10.1136/qshc.2006.022046. 17301192 PMC2464920

[23] Berwick DM . The science of improvement. JAMA. 2008;299:1182-1184. doi:10.1001/jama.299.10.1182 18334694

[24] Baily MA Bottrell M Lynn J , et al. The ethics of using QI methods to improve health care quality and safety. Hastings Cent Rep. 2006;36:S1-S40. doi:10.1353/hcr.2006.0054. 16898359

[25] Goldstein CE Weijer C Brehaut JC , et al. Accommodating quality and service improvement research within existing ethical principles. Trials. 2018;19:334. doi:10.1186/s13063-018-2724-2 29941000 PMC6019798

[26] Ethics GoC-PoR. Tri Council Policy Statement 2 (2018)–Chapter 1: Introduction. https://ethics.gc.ca/eng/tcps2-eptc2_2018_chapter1-chapitre1.html. Published 2018. Accessed February 10, 2022.

[27] Ethics GoC-PoR. Tri Council Policy Statement 2 (2018)–Chapter 9: Research Involvign the First Nations, Inuit and Metis People of Canada. https://ethics.gc.ca/eng/tcps2-eptc2_2018_chapter9-chapitre9.html. Published 2018. Accessed February 10, 2022.

[28] Fiscella K Tobin JN Carroll JK , et al. Ethical oversight in quality improvement and quality improvement research: new approaches to promote a learning health care system. BMC Med Ethics. 2015;16:63. doi:10.1186/s12910-015-0056-2 PMC4574354 26383770

[29] Flaming D Barrett-Smith L Brown N Corcoran J . “Ethics? But it’s only quality improvement!.” Healthc Q. 2009;12(2):50-55. doi:10.12927/hcq.2009.20661. 19369811

[30] University of Ottawa. Is your project research or quality improvement? guideline & checklist. http://www.ohri.ca/ohsn-reb/. Published 2016. Accessed June 22, 2021.

[31] Vancouver Coastal Health, University of British Columbia. When does your project warrant review by a research ethics board? https://www.vchri.ca/when-does-your-project-warrant-review-research-ethics-board. Published 2012. Accessed September 11, 2021.

[32] Hagen B O’Beirne M Desai S Stingl M Pachnowski CA Hayward S . Innovations in the ethical review of health-related quality improvement and research: the Alberta Research Ethics Community Consensus Initiative (ARECCI). Healthc Policy. 2007;2(4):e164-e177. 19305726 PMC2585461

[33] Memorial University. Does your administrative research project require review by the IAPP Office? https://rpresources.mun.ca/triage/does-your-administrative-research-project-require-review-by-the-iapp-office. Published 2021. Accessed September 11, 2021.

[34] Dalhousie University. Guidelines for differentiating among research, program evaluation and quality improvement. https://www.dal.ca/dept/research-services/responsible-conduct-/research-ethics-/faqs.html. Published 2021. Accessed October 30, 2021.

[35] McGill University Health Centre. Research vs quality initiative screening tool. https://muhc.ca/sites/default/files/users/user181/QI%20vs%20Research%20screening%20tool-FINAL.doc. Published 2021. Accessed September 11, 2021.

[36] Laval University. Conditions Menant A Conclure Qu’Une Approbation Ethique N’est Pas Requise. https://www.cerul.ulaval.ca/wp-content/uploads/2021/05/Derogation-projet-professeurs.pdf. Published 2021. Accessed November 17, 2021.

[37] Queen’s University. Research ethics. https://www.queensu.ca/vpr/ethics. Published 2021. Accessed November 8, 2021.

[38] St. Joseph’s Healthcare MU. Research vs quality improvement. https://www.stjoeshealth.org/about-us/institutional-review-board/st-joseph-mercy-ann-arbor/research-vs-quality-improvement. Published 2014. Accessed September 11, 2021.

[39] University W. Human ethics—frequently asked questions. https://www.uwo.ca/research/ethics/human/faq.html. Published 2021. Accessed November 8, 2021.

[40] Women’s College Hospital. Women’s College Hospital Ethics Assessment Process for Quality Improvement Projects (WCH APQIP): applicant information. https://www.womenscollegehospital.ca/assets/pdf/wihv/WCH_APQIP_ApplicantInformation.pdf. Published 2014. Accessed September 11, 202.

[41] Northern Ontario School of Medicine. Research ethics/animal care. https://www.nosm.ca/research/research-services/research-ethics-animal-care/. Published 2021. Accessed October 30, 2021.

[42] University of Manitoba. Research requiring ethics review. https://www.umanitoba.ca/research/orec/ethics_medicine/requiring_review.html. Published 2021. Accessed November 8, 2021.

[43] University of Saskatchewan. Research vs quality improvement/quality assurance. https://medicine.usask.ca/documents/research/sca-research-vs-quality-improvement-quality-assurance-information.pdf. Published 2019. Accessed November 8, 2021.

[44] University of Calgary. University of Calgary research application. https://cumming.ucalgary.ca/mdprogram/faculty/ume-research-application. Published 2021. Accessed November 8, 2021.

[45] University of Alberta. Guidelines for differentiating among research, program evaluation and quality improvement. uab.ca/reo. Published 2020. Accessed September 11, 2021.

[46] Nunavut Research Institute. Health research in Nunavut special considerations for remote data collection. https://www.nri.nu.ca/health-research-nunavut-special-considerations-remote-data-collection. Published 2020. Accessed September 11, 2021.

[47] William Osler Health System. Research ethics board frequently asked questions. https://www.williamoslerhs.ca/en/research-and-outreach/research-ethics-board-frequently-asked-questions.aspx. Published 2021. Accessed November 17, 2021.

[48] Winnipeg Regional Health Authority. Research ethics. https://wrha.mb.ca/research/ethics/. Published 2021. Accessed November 8, 2021.

[49] Lindenauer PK Benjamin EM Naglieri-Prescod D Fitzgerald J Pekow P . The role of the institutional review board in quality improvement: a survey of quality officers, institutional review board chairs, and journal editors. Am J Med. 2002;113(7):575-579. doi:10.1016/s0002-9343(02)01250-0. 12459404

